# Impact of diabetes and early revascularization on the need for late and repeat procedures

**DOI:** 10.1186/s12933-018-0669-0

**Published:** 2018-02-05

**Authors:** Ady Orbach, David A. Halon, Ronen Jaffe, Ronen Rubinshtein, Basheer Karkabi, Moshe Y. Flugelman, Barak Zafrir

**Affiliations:** 1 Department of Cardiovascular Medicine, Lady Davis Carmel Medical Center, 7 Michal St., 3436212 Haifa, Israel; 2 The Ruth and Bruce Rappaport Faculty of Medicine, Technion-Israel Institute of Technology, Haifa, Israel

**Keywords:** Diabetes mellitus, Cardiac catheterization, Coronary revascularization, Percutaneous coronary intervention

## Abstract

**Background:**

Coronary artery disease often progresses more rapidly in diabetics, but the integrated impact of diabetes and early revascularization status on late or repeat revascularization in the contemporary era is less clear.

**Methods:**

Coronary angiography was performed in 12,420 patients between the years 2000–2015 and early revascularization status [none, percutaneous coronary intervention (PCI) or bypass surgery (CABG)] was determined. Subsequent revascularization procedures were recorded over a median follow-up of 67 months and its relation to diabetic and baseline revascularization status was studied.

**Results:**

Early revascularization status was none in 5391, PCI in 5682 and CABG in 1347 patients. Late revascularization rates were 10, 26 and 11.1% respectively. Diabetes was present in 37%; a stepwise relationship of diabetic status with late revascularization was observed: no diabetes (reference) 14.4%, non-insulin treated diabetes 21% (adjusted HR 1.35, 95% CI 1.23–1.49, p < 0.001) and insulin-treated diabetes 32.8% (adjusted HR 2.20, 95% CI 1.91–2.54, p < 0.001), which was similar in magnitude for each early revascularization state (none, PCI or CABG). Further revascularizations (≥ 2) were also significantly more common in diabetics, in particular if insulin-treated. Glycosylated hemoglobin level was moderately associated with late revascularization in diabetics after early PCI but not following diagnostic catheterization or CABG.

**Conclusions:**

Diabetic status graded by treatment, and in particular insulin therapy, is a strong predictor for late or repeat revascularization irrespective of early revascularization status. The high rate of repeat revascularization in diabetics following PCI remains a challenging issue.

**Electronic supplementary material:**

The online version of this article (10.1186/s12933-018-0669-0) contains supplementary material, which is available to authorized users.

## Introduction

Diabetes mellitus is associated with a more complex, rapidly progressive and diffuse atherosclerosis, leading to unfavorable clinical outcomes and increased risk for post-procedural complications and need for repeat revascularization [1, 2]. However, the extent of coronary artery disease (CAD) differs widely amongst diabetics and a meta-analysis has shown that the increased rate of repeat revascularization in diabetics compared to non-diabetics was no longer seen in patients with non-complex, localized disease [3]. Whatever the relation of diabetic to non-diabetic CAD outcomes, there is growing evidence that patients with stable CAD derive limited benefit from percutaneous coronary intervention (PCI) procedures [4, 5] and randomized trials in which diabetics have been screened for early CAD, with intervention as indicated, have not demonstrated improved outcomes [6].

The role and preferred method of revascularization in diabetics when necessary continues to be debated, and multiple randomized controlled trials have compared revascularization strategies in patients with diabetes [7, 8]. Examination of the need for late or repeat revascularization procedures after early therapeutic triage may shed light on this discussion and in particular a comparison of late revascularization rates in relation to prior revascularization status may demonstrate if early revascularization by PCI for localized disease is beneficial or might be delayed until an increasing extent of disease requires surgical intervention. In addition to the impact of early revascularization we examined the impact of diabetic status and baseline glycemic control on the need for late revascularization in this symptomatic diabetic cohort referred for coronary angiography after the year 2000.

## Methods

### Study population

Retrospective analysis of the cardiac catheterization laboratory database at Carmel Medical Center, Haifa, Israel, between the years 2000 and mid-2015 was performed. During that period 14,337 patients were referred for coronary angiography for the assessment and/or treatment of CAD. Of them, 1917 patients with previous coronary interventions before the year 2000 were excluded from the study, and final study population included 12,420 patients. Only the first cardiac catheterization of each patient during the study period was included, and the immediate revascularization status within 2 months recorded and classified as (a) none (diagnostic catheterization), (b) PCI or (c) referral for coronary bypass surgery (CABG). The presence of diabetes mellitus and its treatment status (non-insulin or insulin therapy) at presentation to cardiac catheterization was prospectively documented by the catheterization team. Data were collected from electronic medical records. Diagnosis of diabetes mellitus was given by primary care physicians according to clinical judgment and customary definitions. Data regarding age, gender, risk factors and comorbidities were also documented. Subsequent coronary revascularization procedures (both PCI and CABG) during follow-up were determined retrospectively from patients’ electronic files and computerized records of health maintenance organizations, and the relation to baseline diabetic and revascularization status was tested.

Glycosylated hemoglobin (HbA1c) values during a peri-procedural period of 6 months from baseline cardiac catheterization were searched for in the electronic database. Out of the diabetic population, 79% had documented baseline HbA1c values, which were divided into tertiles [low tertile (T1, HbA1c ≤ 6.8%), mid tertile (T2, 6.8% < HbA1c ≤ 8%) and high tertile (T3, HbA1c > 8%)].

The study was approved by Carmel Medical Center Ethics Committee with waiving of the need for individual patient consent.

### Data analysis

Continuous data are presented as mean ± standard deviation or median with interquartile range (IQR) and categorical variables as numbers and percentages. Analysis of variance or independent samples *t* test was used to compare continuous variables and Chi square to compare categorical variables. Long-term revascularization rates in relation to baseline revascularization and diabetes status were calculated using the Kaplan–Meier method, and statistical comparison performed using the log-rank test. Multivariate analysis of the association of diabetes status with long-term coronary revascularization was performed using the Cox proportional hazards model with forward stepwise selection of covariates, calculating hazard ratios (HR) and 95% confidence intervals (CI). Included in the models were variables that were statistically significant in a univariate analysis. Additional models taking into account both diabetes status and baseline catheterization procedure were performed.

The association of HbA1c levels of diabetics at index catheterization with future coronary revascularization was investigated. Receiver operating characteristic (ROC) curves along with the area under the curve (AUC) and corresponding 95% CI were constructed for evaluating the value of HbA1c in discrimination of future coronary revascularization. Youden’s Index was used to find an optimal threshold value of HbA1c from the ROC curve.

The results were considered statistically significant when the 2-sided p-value was < 0.05. SPSS statistical software version 20.0 and MEDCALC version 16.8.4 were used to perform all statistical analyses.

## Results

The study included 12,420 patients (6576 with acute coronary syndrome) undergoing their first cardiac catheterization during the study period. Mean age was 64 ± 12 years (71% males) and 4384 (35%) were diabetic (262 diet/exercise treated, 3342 orally-treated and 780 insulin-treated diabetes) (Table 1). The index revascularization status following angiography was none, n = 5391 (Group A), PCI, n = 5682 (Group B) and CABG, n = 1347 (Group C). Patients referred to CABG were older, included a higher proportion of men and had higher rates of diabetes and peripheral vascular disease but lower body mass index (BMI) (Table 1).

**Table 1:** Patient characteristics according to index revascularization status

Variable	Total (n = 12,420)	No revascularization (n = 5391)	PCI (n = 5682)	CABG (n = 1347)	p value
Age (years)	64 ± 12	63 ± 12	65 ± 12	66 ± 10	< 0.001
Male	8875 (71.5%)	3501 (65%)	4317 (76%)	1057 (79%)	< 0.001
BMI (kg/m^2^)	28.2 ± 4.6	28.6 ± 4.9	28.0 ± 4.3	27.8 ± 4.4	< 0.001
Hypertension	8484 (68.3%)	3489 (64.7%)	4026 (70.9%)	969 (71.9%)	< 0.001
Hyperlipidemia	8300 (66.8%)	3354 (62.2%)	4034 (71%)	912 (67.7%)	< 0.001
Family history	2287 (18.4%)	991 (18.4%)	1072 (18.9%)	224 (16.6%)	0.163
Current smoker	2851 (23%)	1189 (22.1%)	1401 (24.7%)	261 (19.4%)	< 0.001
Diabetes mellitus	4384 (35.3%)	1781 (33%)	2042 (35.9%)	561 (41.6%)	< 0.001
Chronic renal failure	633 (5.1%)	242 (4.5%)	322 (5.7%)	69 (5.1%)	0.019
Creatinine (mg/dl)	1.05 ± 0.70	1.04 ± 0.74	1.06 ± 0.68	1.04 ± 0.58	0.285
Hemoglobin (g/dl)	13.6 ± 1.6	13.6 ± 1.6	13.7 ± 1.6	13.6 ± 1.6	< 0.001
PVD	405 (3.3%)	123 (2.3%)	221 (3.9%)	61 (4.5%)	< 0.001
Total cholesterol (mg/dl) (n = 7585)	185 ± 43	184 ± 41	186 ± 44	186 ± 46	0.15
Triglycerides (mg/dl) (n = 7526)	170 ± 116	165 ± 103	177 ± 130	160 ± 94	< 0.001

*BMI* body mass index, *CABG* coronary artery bypass graft surgery, *PCI* percutaneous coronary intervention, *PVD* peripheral vascular disease

**Fig. 1:**
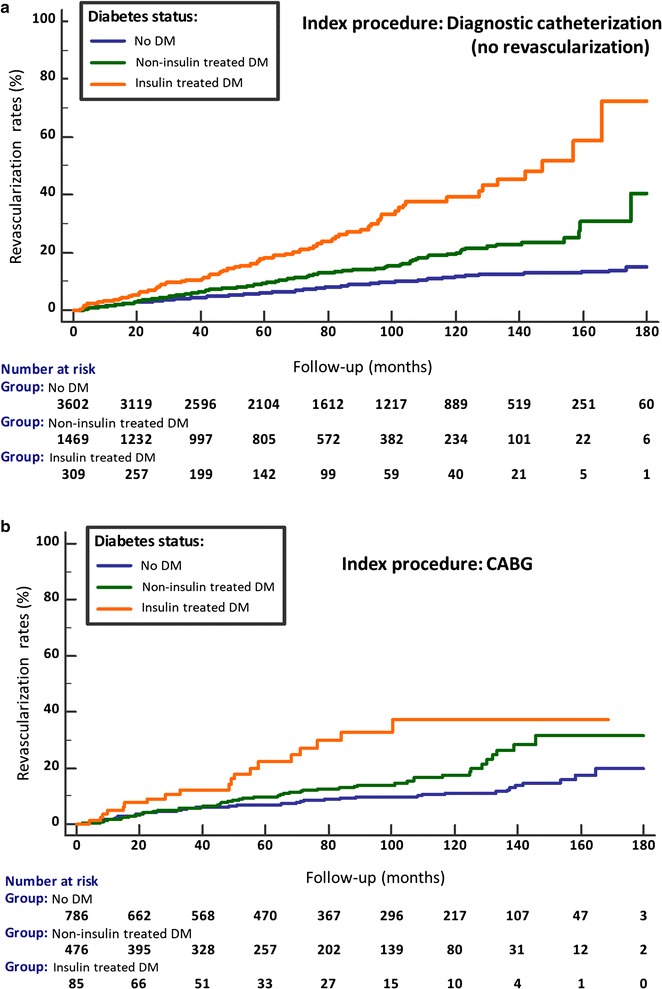
Cumulative revascularization rates according to baseline coronary procedure and diabetes status. Index procedure: **a** diagnostic catheterization (no revascularization); **b** CABG. *CABG* coronary artery bypass graft surgery, *DM* diabetes mellitus, *PCI* percutaneous coronary intervention

**Fig. 2:**
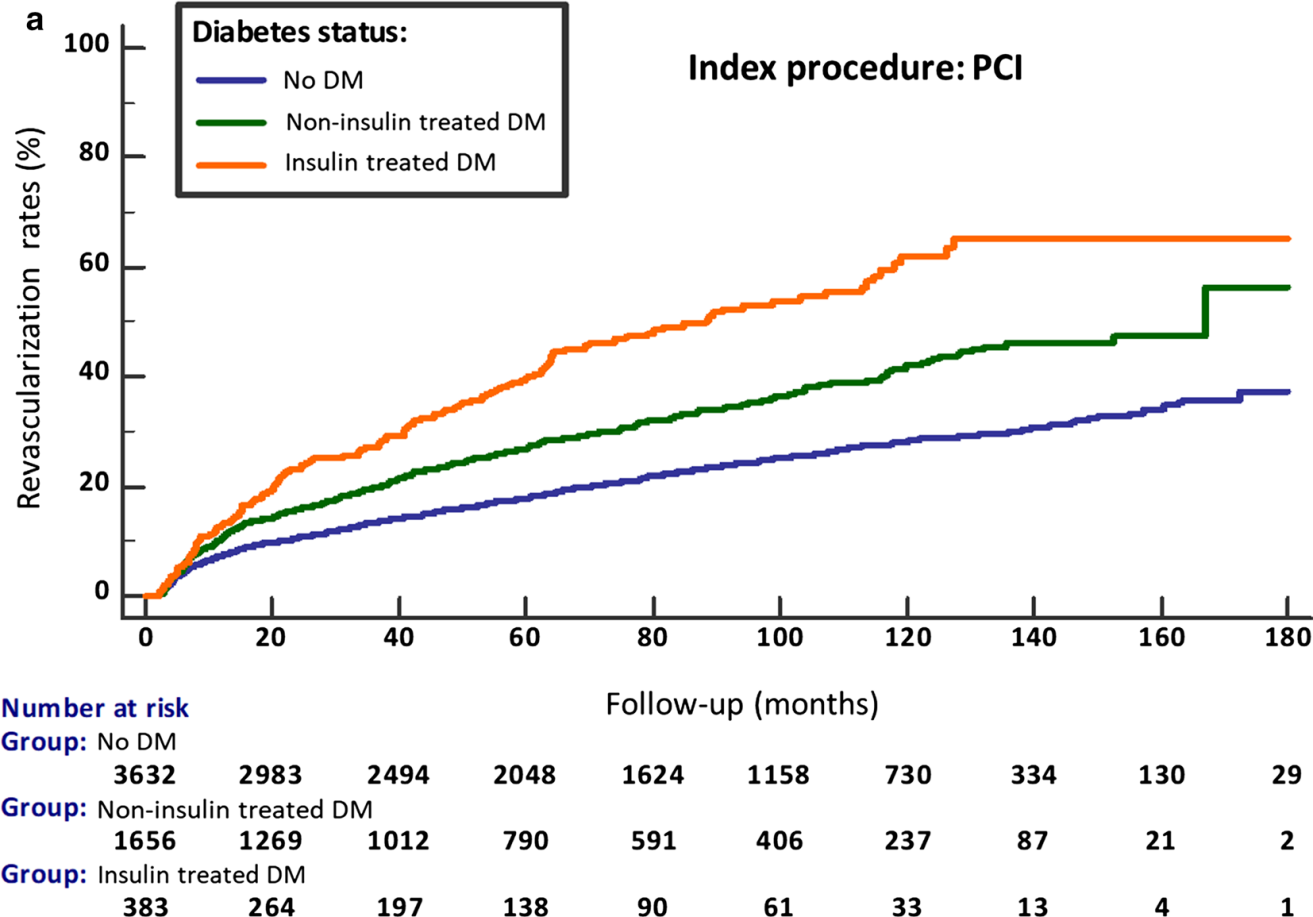
Cumulative revascularization rates according to baseline coronary procedure and diabetes status. Index procedure: **a** PCI. *CABG* coronary artery bypass graft surgery, *DM* diabetes mellitus, *PCI* percutaneous coronary intervention

**Fig. 3:**
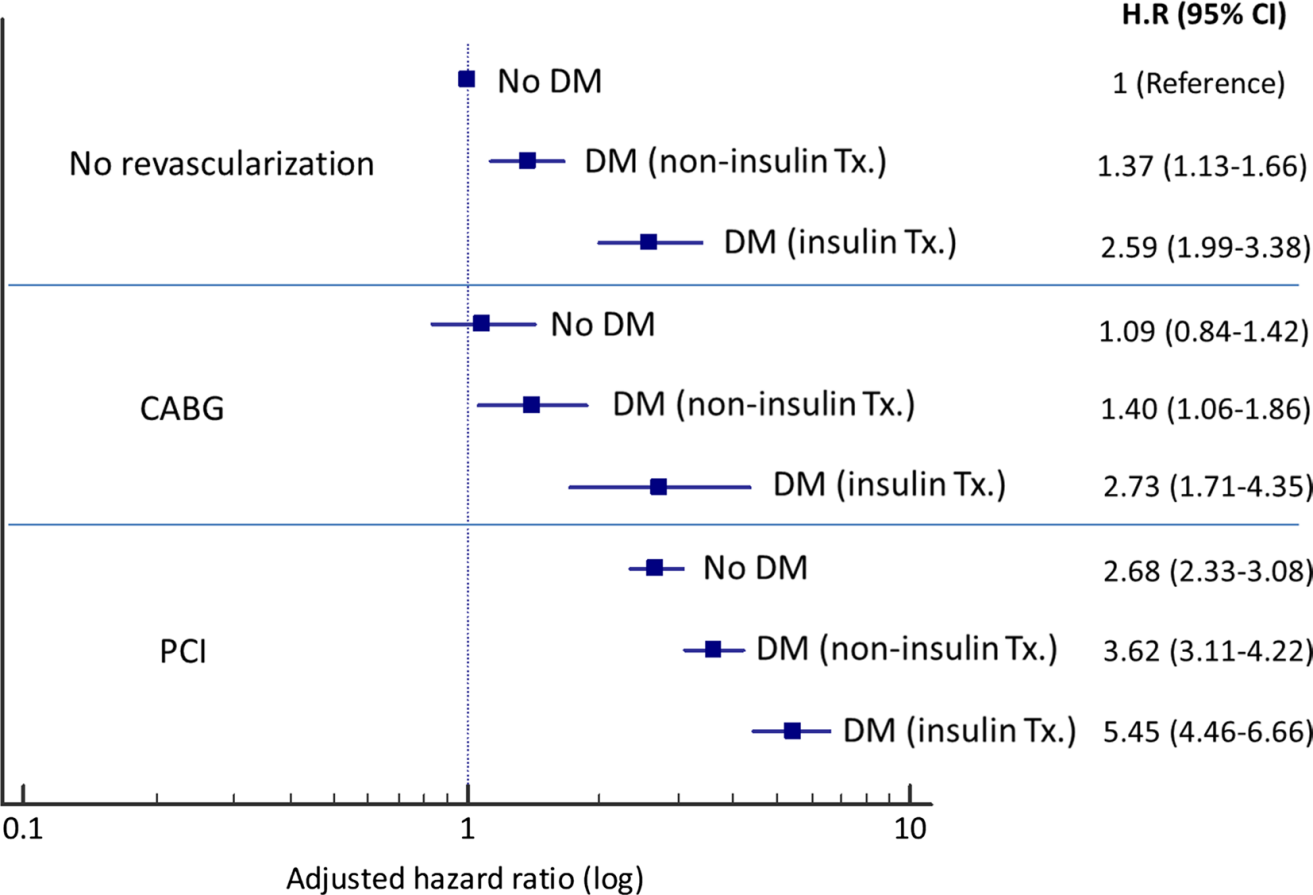
Adjusted hazard ratios for late or repeat revascularization according to index coronary procedure and diabetes status. *Adjustment for: age, gender, BMI, hypertension, hyperlipidemia, renal failure, smoking. *CABG* coronary artery bypass graft surgery, *DM* diabetes mellitus, *HR* hazard ratio, *PCI* percutaneous coronary intervention

**Fig. 4:**
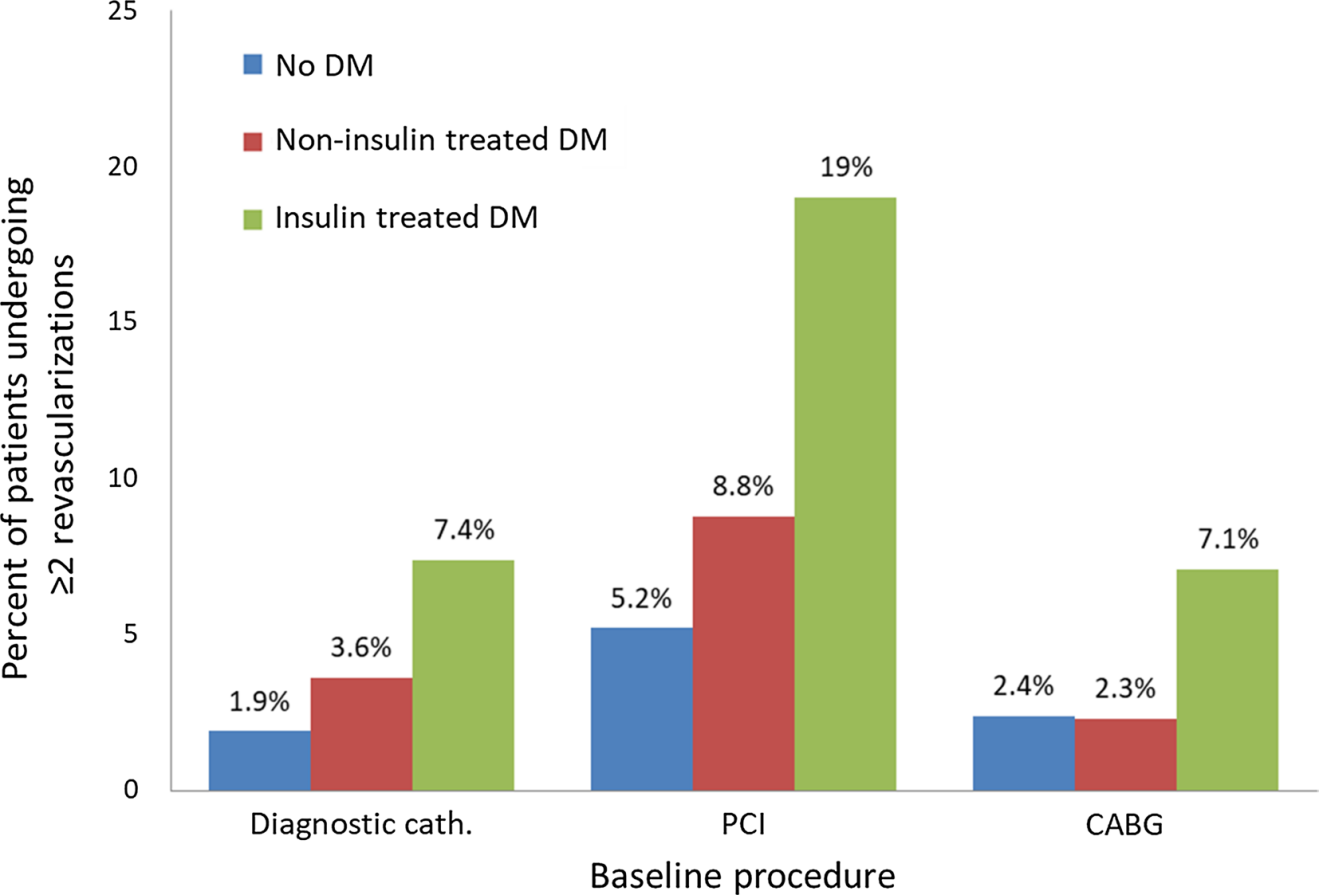
Rate of repeat revascularization (≥ 2 procedures) in relation to index procedure and diabetes status. *CABG* coronary artery bypass graft surgery, *Cath.* catheterization, *DM* diabetes mellitus, *PCI* percutaneous coronary intervention

Over the median follow-up of 67 months (IQR 30-110), crude revascularization rates (PCI or CABG) were 10% in Group A, 26% in Group B and 11.1% in Group C. A stepwise increase in the rates of revascularization over time, from no diabetes to non-insulin and insulin treated diabetes, was demonstrated in each of the 3 baseline cohorts (p < 0.001; Figs. 1a, b, 2a). This was observed also after multivariate adjustment (Fig. 3). In the overall study population, non-diabetics had 14.4% late revascularizations during follow-up, non-insulin treated diabetics 21%, while 32.8% of the insulin-treated diabetics underwent revascularization during follow-up. The progressive increase in the risk ratio for late revascularization across diabetes presence and treatment status was not attenuated significantly in the overall cohort, after additional adjustment for the index revascularization status or presentation with acute coronary syndrome (Table 2); compared to non-diabetics, non-insulin treated diabetics had an adjusted HR of 1.35 (95% CI 1.23–1.49), p < 0.0001 and insulin treated diabetics had an adjusted HR of 2.20 (95% CI 1.91–2.54), p < 0.001. Further revascularizations (≥ 2 repeat procedures) were also significantly more common in diabetics versus non-diabetics, in particular if insulin-treated (7.4% versus 1.9% in the no revascularization group, 19% versus 5.2% in the PCI group and 7.1% versus 2.4% in the CABG group) (Fig. 4).

**Table 2:** Adjusted hazard ratios for coronary revascularization during follow-up, according to diabetic status

	Age and gender adjustment	Multivariate adjustment^a^	Multivariate + acute coronary syndrome adjustment^b^	Multivariate + index procedure adjustment^c^
No diabetes (n = 8036)	1 (reference)	1	1	1
Non-insulin treated diabetes (n = 3604)	1.69 (1.54–1.85) p < 0.001	1.34 (1.22–1.48) p < 0.001	1.33 (1.21–1.46) p < 0.001	1.35 (1.23–1.49) p < 0.001
Insulin treated diabetes (n = 780)	3.05 (2.66–3.49) p < 0.001	2.25 (1.96–2.60) p < 0.001	2.22 (1.93–2.56) p < 0.001	2.20 (1.91–2.54) p < 0.001

^a^Adjustment for: age, gender, BMI, hypertension, hyperlipidemia, creatinine level and smoking

^b^Acute coronary syndrome (n = 6576) versus non acute coronary syndrome (n = 5844)

^c^Index procedure: (a) diagnostic catheterization (no revascularization), (b) CABG, (c) PCI

Amongst the diabetic population, 3443 (79%) had HbA1c levels documented within 6 months from index coronary catheterization. Mean HbA1c levels were 7.66 ± 1.58%. HbA1c levels of the diabetic patients were divided into tertiles. After adjustment for age, gender, hypertension, hyperlipidemia, renal failure, smoking and index catheterization group, the proportional hazard ratio for the need of future coronary revascularization during follow-up was 1.06 (95% CI 0.88–1.28), p = 0.517 for patients in the mid tertile of HbA1c (6.8% < T2 ≤ 8%) and 1.34 (95% CI 1.12–1.61), p = 0.002 for those in the highest tertile of HbA1c (T3 > 8%), compared to the lowest tertile (T1 ≤ 6.8%, reference group). Further assessment of revascularization rates was performed in each of the index catheterization groups, according to an HbA1c cutoff of 8%, corresponding to the upper HbA1c tertile. Of those diabetics undergoing baseline diagnostic catheterization, 12.4% had coronary revascularization during follow-up in the HbA1c ≤ 8% group versus 14.1% in the HbA1c > 8% group, p = 0.429; in diabetic patients that underwent PCI, 26.3% had future revascularizations in the HbA1c ≤ 8% group versus 34.7% in HbA1c > 8%, p < 0.001, whereas in patients in whom CABG was performed, late revascularization rates were 10.4% versus 13.9%, respectively, p = 0.280. The corresponding Kaplan–Meier curves are presented at Additional file 1: Figure S1a–c. The ability of HbA1c to discriminate between those needing or not future revascularization in diabetics was low (AUC 0.540, 95% CI 0.523–0.557, p = 0.0013). The optimal threshold value of HbA1c was 7.91% (sensitivity 40%, specificity 68%).

## Discussion

In the study cohort referred for coronary angiography after the year 2000, diabetes was associated with a long-term risk for late or repeat revascularization which increased progressively with the treatment status of diabetes. Although overall revascularization rates were significantly higher in patients undergoing PCI, the stepwise impact of diabetes status on future revascularization was similar in patients triaged at the index assessment to medical therapy only, PCI or CABG. Insulin treatment in particular was associated with worse outcomes, as demonstrated by 3–4 times higher rates of repeat (≥ 2) revascularization during follow-up compared to those without diabetes, regardless of baseline angiographic findings, whereas HbA1c levels had relatively low discriminative value for predicting future coronary revascularization.

It is interesting that patients undergoing CABG, who presumably had overall more diffuse or extensive disease than those treated with PCI or those not revascularized at all, had a repeat revascularization rate similar or less than those not undergoing any revascularization, while patients undergoing PCI had a much higher rate of revascularization. These findings raise the question if a more conservative approach to PCI in diabetics might currently be more advantageous, employing novel anti-hypoglycemic and lipid lowering therapies and intervening only surgically when disease becomes more extensive.

The interaction between diabetes, atherogenesis and adverse cardiovascular outcomes is multifactorial [9, 10]. Diabetes is associated with glycemic and metabolic derangements, increased platelet aggregation, hypercoagulability and a prothrombotic milieu. Endothelial dysfunction, adverse vascular effects of advanced glycation end products and systemic inflammation all promote atherothrombosis in the setting of diabetes [11]. Diabetics have higher prevalence of a more extensive and multivessel CAD and increased plaque burden which is more vulnerable to rupture. The persistent impact of these adverse effects gives rise to increased risk for acute coronary syndromes, impaired collateral development and increase in post-procedural complications including restenosis, acute thrombosis and the need for repeat coronary revascularizations [1, 12]. Studies have shown that a significant part of repeat revascularization procedures in diabetics result from progression of atherosclerosis in a different vessel or remote segment from the one previously treated, and that non-culprit lesion progression is particularly common in diabetics after acute coronary syndromes [13, 14].

In the current study, diabetes treatment intensity and especially insulin therapy was a marker of increased long-term risk for coronary revascularization, regardless whether catheterization was diagnostic only or resulted in PCI or CABG. Furthermore, insulin therapy was highly associated with more repeat (≥ 2) revascularizations. It is possible that insulin treatment reflects a more prolonged and advanced stage of diabetes. Studies have shown that a longer duration of diabetes and poorer glycemic control are associated with plaque phenotype with increased thin-cap fibroatheroma, associated with risk of rupture and acute coronary events [15]. Although we could not adjust diabetes status for duration of the disease, multivariate adjustment was made for baseline characteristics associated with diabetes severity. In addition, the correlation between revascularization in diabetics and baseline HbA1c levels was less pronounced than with insulin treatment. The cardiovascular safety of exogenous insulin therapy in type 2 diabetes is debated. Possible adverse effects of insulin therapy include hypoglycemia which may promote adrenergic discharge, fluid retention and electrolyte imbalance, increasing the risk for life-threatening arrhythmias [16]. In addition, iatrogenic hyperinsulinemia can promote pro-inflammatory responses and stimulate hormonal over-activation and was suggested to be associated with endothelial and platelet dysfunction in diabetes [17, 18].

Multiple randomized controlled trials have compared revascularization strategies in patients with diabetes [7]. Drug eluting stent implantation has reduced the rates of restenosis and the need for repeat revascularization in diabetics compared to bare metal stents [19–21]. However, outcomes in general are still inferior to those in non-diabetics, and diabetes remains a significant risk factor for adverse clinical events after stent implantation, even with the newer-generation drug eluting stents [22–24], although this may not be the case in all sub-groups [3]. Moreover, insulin treatment by itself was shown to be associated with significantly worse cardiovascular outcomes after PCI compared to non-insulin treated diabetes [25, 26]. Nevertheless, compared to bare metal stents, drug eluting stents were shown to be associated with a significantly lower rate of repeat revascularization, without any increase in myocardial infarction or mortality in patients with insulin treated diabetes, during a follow up period of 1 year [27].

In accordance with the current study results, revascularization trials have generally shown considerably higher rates of repeat revascularization procedures after PCI compared to CABG, with a relative risk which increased in diabetics [28]. The Coronary Artery Revascularization in Diabetes (CARDia) trial displayed significantly higher rates of further revascularizations at 1 year after PCI compared to CABG in diabetics with mutlivessel CAD, while similar death rates and lower rates of non-fatal stroke were observed in those undergoing PCI [29]. Interestingly, major adverse cardiovascular events (death, myocardial infarction, stroke and repeat revascularization) were similar in insulin versus non-insulin treated diabetics undergoing CABG, in contrast to significantly higher event rates in insulin versus non-insulin treated diabetics undergoing PCI. This is in contrast to our results showing significantly higher rates of repeat revascularizations during follow-up in insulin treated versus non-insulin treated diabetic patients after both PCI and CABG.

The 5-year results of the SYNTAX trial, which compared CABG surgery with PCI for the treatment of patients with left main coronary disease or three-vessel disease, showed significantly increased rates of repeat revascularization with PCI (25.9%) versus CABG (13.7%) at 5 years, very similar to our current results [30, 31]. The gap widened with the increase in the SYNTAX score, a sign of complexity of the CAD, commonly observed in diabetics. No subgroup analysis was provided for the quarter of the study population which had diabetes. In the Future Revascularization Evaluation in Patients with Diabetes Mellitus: Optimal Management of Multivessel Disease (FREEDOM) Trial, the researchers evaluated whether aggressive medical therapy and the use of drug-eluting stents could alter the revascularization approach for patients with diabetes and multivessel CAD [32]. CABG was superior to PCI, reducing rates of death and myocardial infarction, with a higher rate of stroke. 1-year repeat revascularization was still significantly higher in the PCI group (12.6%) compared to the CABG group (4.8%).The composite endpoint was not affected by the baseline HbA1c level (cutoff of 7%). However, a subgroup analysis documented that insulin-treated patients had worse clinical outcome regardless of the treatment arm [33]. Moreover, the 1-year repeat revascularization rate was higher (HR 1.44, 95% CI 1.05–1.97) in insulin treated diabetes, primarily due to increased repeat revascularization procedures in the insulin-treated PCI group. This is in line with the current study results, showing that insulin treatment at baseline is a far stronger risk marker for revascularization than HbA1c levels in diabetics.

Overall, our findings are consistent with previous reports from studies displaying considerably higher rates of revascularizations among diabetic patients undergoing PCI versus CABG, which is the main driver for the differences observed in composite outcomes of major adverse cardiovascular events between the 2 revascularization strategies [34]. Although PCI with new generation drug eluting stents has probably narrowed the gap with surgery, the far greater need for subsequent revascularization after PCI remains a challenge in current interventional cardiology, especially in insulin treated diabetics [35].

### Study limitations

Several limitations of this study should be noted. This is a single center study, and therefore may not reflect the clinical practice of other institutions. We did not have information regarding CAD anatomy and accordingly did not assess target vessel revascularization nor the prognostic impact of single compared to mulitvessel or left main CAD. In addition, we did not have data on left ventricular ejection fraction or the presence of chronic heart failure, which may have been a predictor for late revascularizations. Moreover, oral hypoglycemic drugs were treated as a single group. Hence, we could not assess the effect of newer antidiabetic agents which have recently shown to improve cardiovascular outcomes and were not available during much of the study period. Due to the retrospective nature of this study, HbA1c levels were not tested adjacent to the baseline coronary procedure in all diabetic patients, and were not available within 6 months from the baseline procedure in about 20% of the diabetic population; this limited the ability to use imputation for missing data, and may have impacted on the prognostic value of HbA1c.

## Conclusions

The presence of diabetes and in particular insulin-treated diabetes is an independent and strong predictor of late and repeat coronary revascularization, in all patients undergoing baseline coronary angiography with or without revascularization. The high rate of repeat revascularization procedures in diabetics following PCI remains a challenging issue.

## Additional file

**Additional file 1: Figure S1.** Cumulative revascularization rates according to baseline coronary procedure and glycosylated hemoglobin level. Index procedure: (a) diagnostic catheterization (no revascularization); (b) CABG; (c) PCI. CABG, coronary artery bypass graft surgery; HbA1c, glycosylated hemoglobin; PCI, percutaneous coronary intervention.

## Abbreviations

AUC: area under curve
CABG: coronary artery bypass grafts surgery
CAD: coronary artery disease
CI: confidence interval
HbA1c: glycosylated hemoglobin
HR: hazard ratio
IQR: interquartile range
PCI: percutaneous coronary intervention
ROC: receiver operating characteristic curve
