# Supplementary material for: Impact of diabetes and early revascularization on the need for late and repeat procedures
Source: Cardiovasc Diabetol. 2018 Feb 5;17:25. doi: 10.1186/s12933-018-0669-0 (PMC5798191; doi:10.1186/s12933-018-0669-0)
Supplement: Supplementary file 1 — Additional file 1: Figure S1. Cumulative revascularization rates according to baseline coronary procedure and glycosylated hemoglobin level. Index procedure: (a) diagnostic catheterization (no revascularization); (b) CABG; (c) PCI. CABG, coronary artery bypass graft surgery; HbA1c, glycosylated hemoglobin; PCI, percutaneous coronary intervention. [file 12933_2018_669_MOESM1_ESM.docx]

**Figure S1: Cumulative Revascularization Rates, According to Baseline Coronary Procedure and Glycosylated Hemoglobin Level**

Index procedure: (S1a) diagnostic catheterization (no revascularization); (S1b) CABG; (S1c) PCI.

CABG, coronary artery bypass graft surgery; HbA1c, glycosylated hemoglobin; PCI, percutaneous coronary intervention

**S1a:**

**
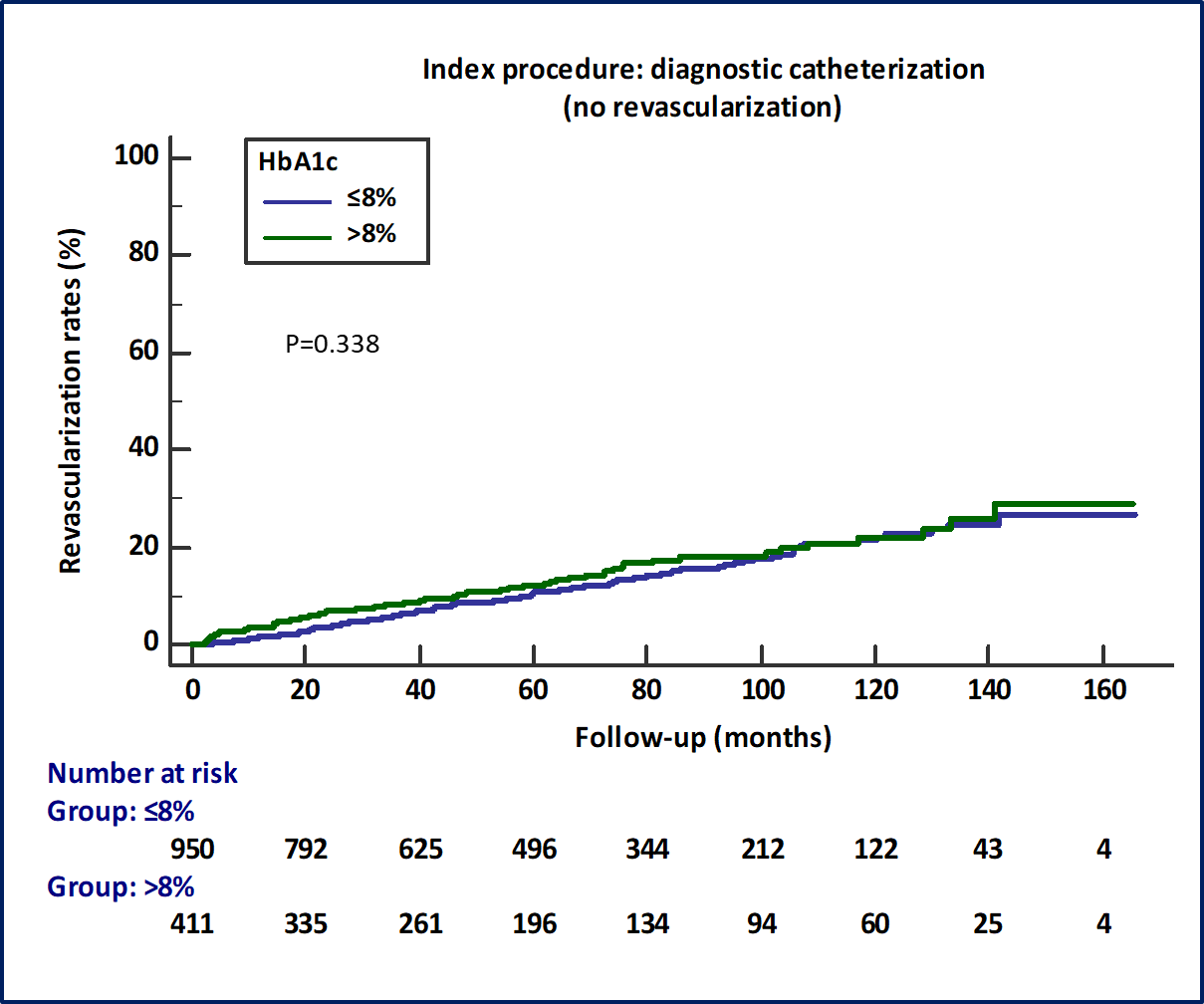
**

**S1b:**

**
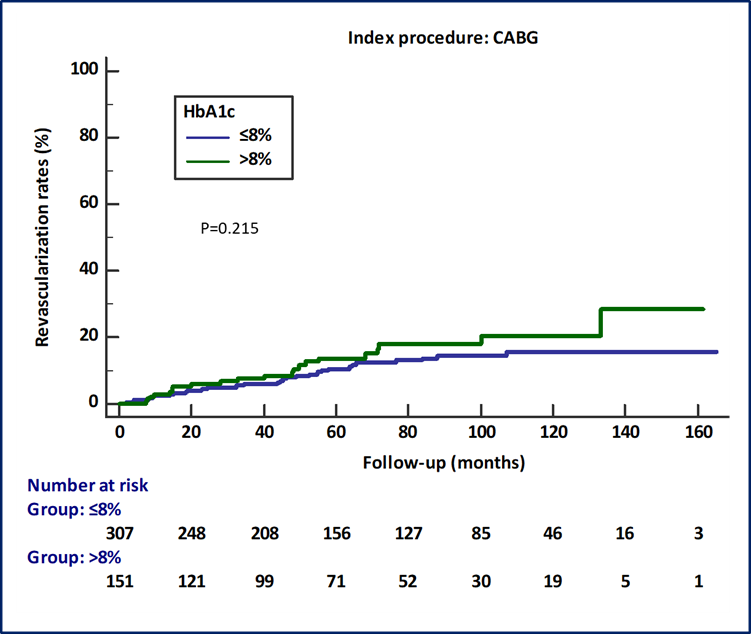
**

**S1c:**

**
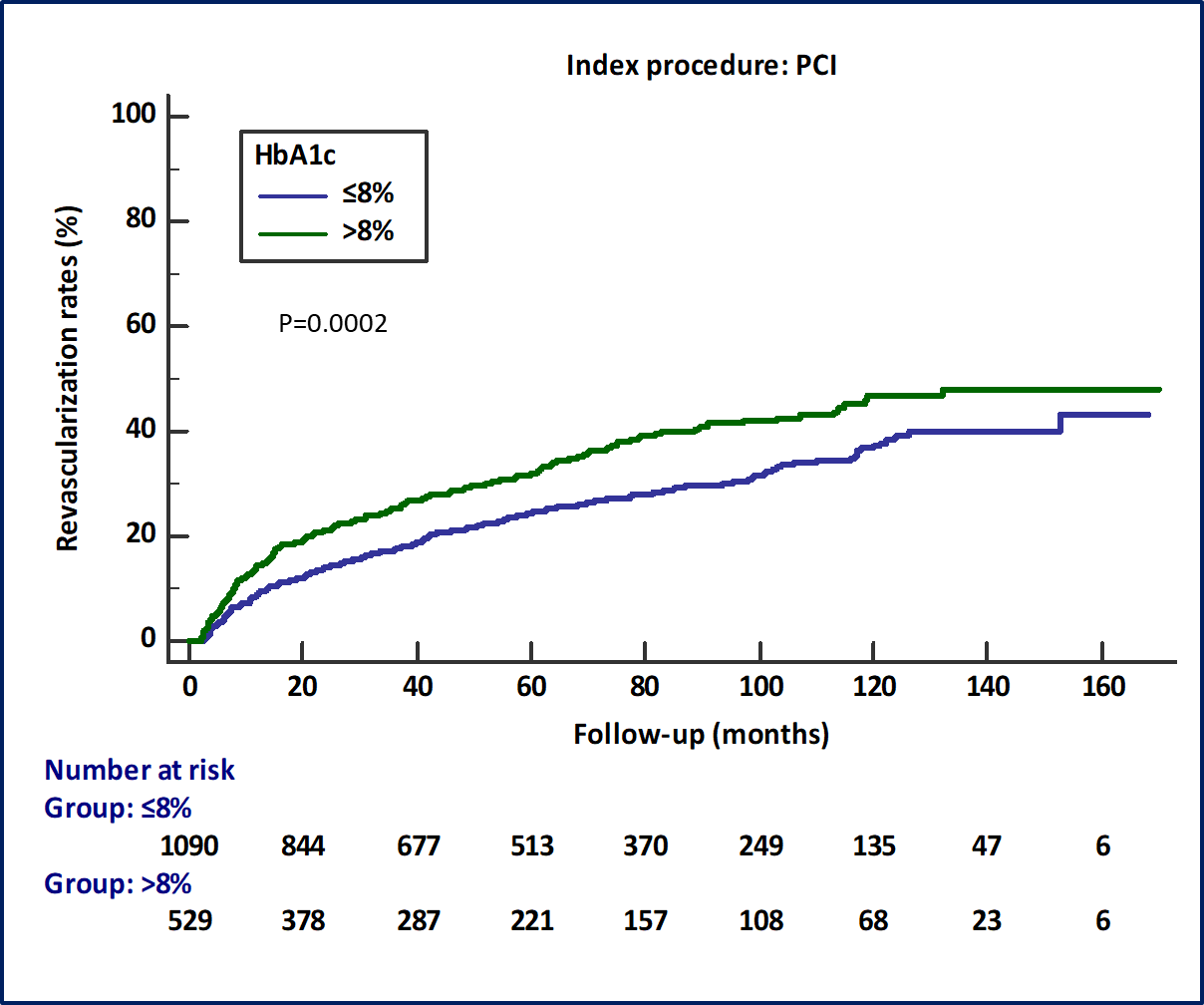
**
